# Acupuncture PC6 for postoperative nausea and vomiting at different times: A protocol for systematic review and meta analysis

**DOI:** 10.1097/MD.0000000000020452

**Published:** 2020-05-29

**Authors:** Kejin Shi, Fengyi He, Ying Tang, Xiao Xiao, Jiayuan Zhang, Yuxia Jin, Yunxia Wang, Qi Zhang

**Affiliations:** Chengdu University of Traditional Chinese Medicine, Chengdu, Sichuan Province, China.

**Keywords:** acupuncture, pC6 point, postoperative nausea and vomiting, protocol, systematic review

## Abstract

**Background:**

Postoperative nausea and vomiting (PONV) is a condition that commonly following anesthesia and surgery, antiemetics can lead to some side effects in treating PONV. Acupuncture PC6(Neiguan) has been widely used in the prevention and treatment of postoperative nausea and vomiting. However, there still exists controversy towards its effectiveness, appropriate, and effective intervention time. We, therefore, design this meta-analysis to assess the effectiveness and confirm the optimal time of acupuncture PC6 point for PONV.

**Methods:**

The following electronic databases will be searched from their inception to April 2020, including PubMed, Cochrane Library, EMBASE, Web of Science, WHO International Clinical Trials Registry Platform, Chinese National Knowledge Infrastructure, WanFang Database, Chinese Biomedical Literature Database, the Chongqing VIP Chinese Science, and Technology Periodical Database. All randomized controlled trials in English or Chinese involving acupuncture PC6 for patients with PONV will be included. Two reviewers will independently responsible for the data extraction, study selection, risk of bias assessment and assessment of study quality. The primary outcome was the number of postoperative nausea, postoperative vomiting and PONV during 0 to 6 hours and after 6 hours of the postoperatively. The secondary outcome is the number of people with side effects and the use of rescue therapy. The meta-analysis will be conducted using RevMan V.5.3.5 statistical software.

**Results:**

This systematic review will evaluate the efficacy and appropriateness time of acupuncture PC6 in the treatment of PONV.

**Conclusion:**

This study will provide high-quality current evidence of the effectiveness and optimal time of acupuncture PC6 point for the patient with PONV.

**Ethics and dissemination:**

Ethical approval is not required; this review will not involve individuals’ information. The results will be published in a peer-reviewed publication or disseminated in relevant conferences.

**INPLASY Registration number:**

DOI 10.37766/inplasy2020.4.0012

## 1 Introduction

Postoperative nausea and vomiting (PONV) is a complication commonly after anesthesia and surgery, with an incidence of 30% to 40%, but as high as 53% to 72% in a part of high-risk patients.^[1]^ In addition to experience discomfort, the complication can also cause dehydration, metabolic disorders, wound dehiscence and risk of pulmonary aspiration.^[2,3]^ These adverse effects also delayed hospital discharge and increase health care costs. Although various antiemetic drugs are widespread use for PONV, they also result in some adverse effects, such as sedation, headache, n extrapyramidal reaction.^[4,5]^ Because of these negative side effects, researchers have started to search for an alternative treatment to prevent and treat PONV.^[6]^

Acupuncture is a minimally-invasive therapeutic which is a component of traditional Chinese medicine. Now many patients and families are interested in using it to control symptoms.^[7]^ In Australia, it has been the fourth preferred complementary therapy in hospitalized patients for treating PONV.^[8]^ Acupuncture is a non-pharmacological therapy for the prevention of PONV.^[9]^ Studies have shown that acupuncture PC6 can effectively reduce the frequency of PONV.^[10]^ Previous systematic reviews studies have focused on evaluating the efficacy and safety of acupuncture PC6 in the treatment of PONV,^[11]^ but different intervention timings may result in different influence.^[12]^ However, acupuncture performed in a conscious patient(before or after anesthesia) or during anesthesia still controversial.^[13–16]^ Recent years there were several RCTs has been published.^[17,18]^ These publications will be potentially conducive to offer existing evidence to re-evaluate the effectiveness of acupuncture PC6 points for patients with PONV.

Meanwhile, we will conduct this systematic review to assess the optimal intervention time of acupuncture PC6 points for patients with PONV. Moreover, we will also compare the effectiveness of acupuncture PC6 point with other treatments, and evaluate the appropriateness duration time of acupuncture to achieve the maximum effect.

## 2 Methods

### 2.1 Study registration

The protocol for this systematic review was registered on INPLASY (Unique ID number), and is available in full on the inplasy.com (https://doi.org/10.37766/inplasy00000000). The registration number is INPLASY202040012. This systematic review protocol report is based on the PRISMA-P guidelines, and will be conducted in accordance with the PRISMA guidelines.

### 2.2 Inclusion criteria for study selection

#### 2.2.1 Type of study

Randomized controlled trials assessing acupuncture therapy for PONV will be eligible for inclusion, and were reported in English or Chinese. No publication status restrictions.

#### 2.2.2 Type of participant

Participants aged 18 years or older with regardless of the type of anesthesia or surgery, risk score for PONV, duration of anesthesia will be included. Regardless of their gender, nationality, education, or economic status.

#### 2.2.3 Type of intervention

The intervention group treatment including manual acupuncture or electroacupuncture, and acupuncture at the point of PC6, without restrictions on unilaterally or bilaterally. Acupuncture combined with drug therapy will be excluded. The control group should accept one of the following treatment methods: conventional drugs, placebo, sham acupuncture.

#### 2.2.4 Types of outcome measures

The primary outcome was the number of postoperative nausea, postoperative vomiting and PONV during 0 to 6 hours and after 6 hours of the postoperatively.

The secondary outcome is the number of people with side effects and the use of rescue therapy.

### 2.3 Search methods

The following electronic databases will be searched from their inception to April 2020, including PubMed, Cochrane Library, EMBASE, Web of Science, WHO International Clinical Trials Registry Platform, Chinese National Knowledge Infrastructure, WanFang Database, Chinese Biomedical Literature Database, the Chongqing VIP Chinese Science and Technology Periodical Database. We will only include RCT trials published in English or Chinese. The search strategy for PubMed is shown in Table 1. Other online databases will be used in the same strategy.

**Table 1:**
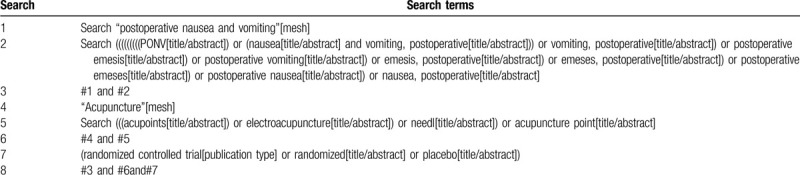
Search strategy used in PubMed.

### 2.4 Data collection and analysis

#### 2.4.1 Selection of studies

The trained reviewers will independently accomplish the following process:

1. All the search results will be imported into Endnote X9.
2. Remove duplicated or ineligible studies.
3. Screen the titles, abstracts, and full texts of all studies according to inclusion criteria to identify eligible trials.
4. Obtain the full text of all eligible studies

**Figure 1:**
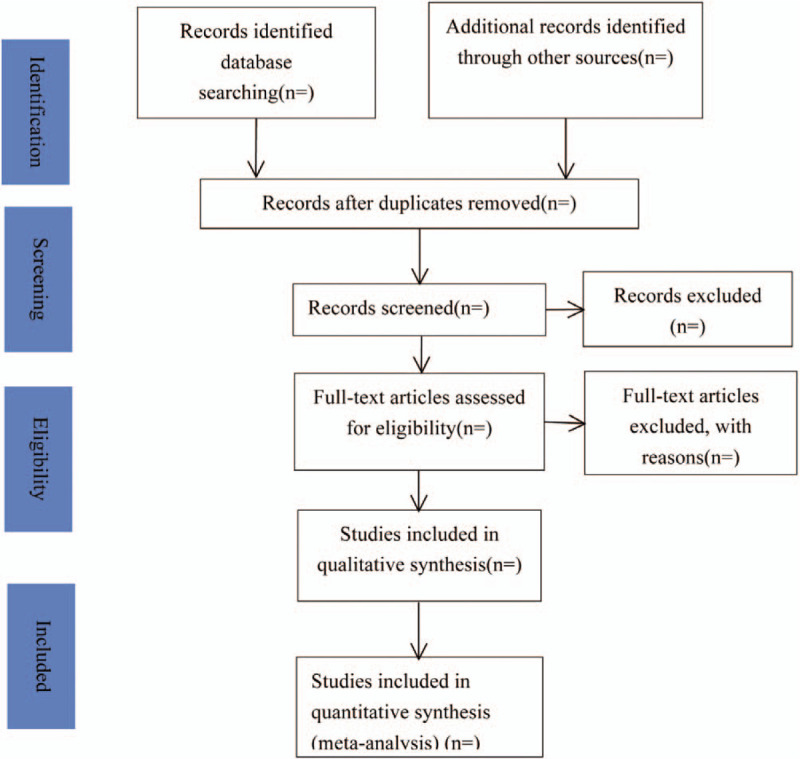
Flow chart of study selection process.

Any different opinions will be resolved by the third reviewer. The study selection process is summarized in a PRISMA flow chart (Fig. 1).

#### 2.4.2 Data collection and management

Two reviewers will extract the following data from the selected studies independently, and any discrepancies will be resolved by negotiation with a third reviewer. The information including: author list, ear of publication, age, ample size, types of surgery, type of control, timing of intervention, duration of intervention, insertion acupoints, depth or frequency of needle insertion, side effects, rescue antiemetic. The Review Manager Software (RevMan V.5.3.5) will be used for analysis and synthesis data.

#### 2.4.3 Assessment of risk of bias in included studies

Two reviewers will independently assess the risk of bias according to the Cochrane Collaboration's tool assessment method. The study bias will be conducted into 3 levels: “low risk”, “high risk”, and “unclear” in accordance with the following criteria: sequence generation, allocation concealment, blinding, incomplete data, selective outcome reporting, and other sources of bias. Any disagreement caused by assessment of the bias, there is a need for resolved with a third reviewer (ZQ).

#### 2.4.4 Measures of treatment effect

The statistical analyses will be performed using the risk ratio with 95% confidence intervals (CIs). Relative risk and odds ratio are used for all dichotomous outcomes data. The weighted mean difference (WMD) or the standard mean difference (SMD) will be conducted using continuous outcomes.

#### 2.4.5 Unit of analysis

The analysis unit will be the individual participant.

#### 2.4.6 Management of missing data

We will contact the correspondent author for more details if basic information is missing. If the missing data is not available, an available case study will be conducted (including all data from known results), and we will perform a sensitivity analysis to evaluate whether the results are inconsistent.

#### 2.4.7 Assessment of heterogeneity

The standard x^2^ test will used to assess the statistical heterogeneity, The I^2^ value is classified into the following 4 categories according to the Cochrane Handbook:

1. 0% to 40%, indicates homogeneous;
2. 30% to 60%, considers moderate heterogeneity;
3. 50% to 90%, suggests substantial heterogeneity;
4. 75% to 100% represents considerable heterogeneity.

We will use meta-regression method to analyze the cause of the heterogeneity if I^2^ is more than 50%.

#### 2.4.8 Assessment of reporting biases

If the numbers of available studies more than 10 trials, funnel plots will be used to assess the reported biases. We will use the Egger test to detect the symmetry of funnel plots.

#### 2.4.9 Data synthesis

RevManV.5.3.5 will be used for data synthesis. The fixed effect model will be used for data synthesis if no substantial statistical is investigated, while the random effects model will be adopted if there is substantial statistical heterogeneity. But if there is significant clinical heterogeneity, we will conduct the subgroup analysis or sensitivity analysis, or the narrative and qualitative summary.

#### 2.4.10 Subgroup analysis and investigation of heterogeneity

Subgroups will be conducted according to optimal timing (preoperative, intraoperative, postoperative, continuous intervention), duration of intervention time. We will perform a further subgroup analysis if the previous analysis indicates considerable heterogeneity.

#### 2.4.11 Sensitivity analysis

Sensitivity analysis will be adopted to evaluate the robustness and reliability of the review results. The sample size, methodological quality, studies design, and missing data will be assessed. Then, we will repeat analyze the data after the exclusion of low methodological quality studies.

#### 2.4.12 Summary of evidence

The quality of evidence will be determined by grading of recommendations assessment, development, and evaluation , and will be classified into high, moderate, low, and very low quality.

## 3 Discussion

PONV is a common complication that has a significantly negative impact on the life quality and recovery of patients. Despite antiemetic drug treatment, they are not certainly effective for all PONV patients, leading to some side effects, acupuncture PC6 point, now as the complementary and alternative therapy has been widely applied in clinical practice. The National Institutes of Health (NIH) 1997 Consensus Statement reported that “promising results have emerged showing the efficacy of acupuncture in adult postoperative”.^[19]^ Although acupuncture increasing popularity as a choice of nonpharmacological treatment nowadays, the mechanism has not been established.

Traditional Chinese medicine theory thinks both qi and blood losing balanced because of surgery or anesthesia. The imbalance state leads to the stomach qi move up, then cause nausea and vomiting. Acupuncture PC6 point can regulate the movement of both qi and blood.^[20,21]^ Previous studies have suggested that that stimulating PC6 point may have an effect on the endocrine system of the body, regulate the level of beta-endorphins in cerebrospinal fluid and the transmission of endogenous opioids and 5-hydroxytryptamine in serum, inhibit the gastric acid secret, regulate gastrointestinal function, and thus stop nausea and vomit.^[22,23]^ In addition, it has been shown that intervention timing is an important variable for its effectiveness.^[21]^ However, optimal intervention timing of acupuncture PC6 point for PONV is still controversial.

Hence, we will analyze the previous RCT trials to determine the appropriate and effective intervention time of acupuncture PC6 for PONV. As far as we know, this study will be the first systematic review to research on this issue. There may be some limitations in this review, we believe that the review will provide important information for clinical doctors and health policymakers.

## Author contributions

**Conceptualization:** Kejin Shi.

**Data curation:** Jiayuan Zhang, Fengyi He.

**Formal analysis:** Xiao Xiao, Jiayuan Zhang, Yuxia Jin.

**Investigation:** Kejin Shi.

**Methodology:** Kejin Shi.

**Project administration:** Kejin Shi, Qi Zhang.

**Software:** Yunxia Wang.

**Supervision:** Yunxia Wang.

**Writing – original draft:** Kejin Shi, Ying Tang.

**Writing – review & editing:** Kejin Shi.
